# Platelet-Rich Plasma-Releasate (PRPr) for the Treatment of Discogenic Low Back Pain Patients: Long-Term Follow-Up Survey

**DOI:** 10.3390/medicina58030428

**Published:** 2022-03-16

**Authors:** Koji Akeda, Norihiko Takegami, Junichi Yamada, Tatsuhiko Fujiwara, Kohshi Ohishi, Satoshi Tamaru, Akihiro Sudo

**Affiliations:** 1 Department of Orthopaedic Surgery, Mie University Graduate School of Medicine, Tsu 514-8507, Japan; n-takegami@clin.medic.mie-u.ac.jp (N.T.); yamada-j@med.mie-u.ac.jp (J.Y.); tatsuhiko-f@med.mie-u.ac.jp (T.F.); a-sudou@clin.medic.mie-u.ac.jp (A.S.); 2 Department of Transfusion Medicine and Cell Therapy, Mie University Hospital, Tsu 514-8507, Japan; koishi@clin.medic.mie-u.ac.jp; 3 Clinical Research Support Center, Mie University Hospital, Tsu 514-8507, Japan; tamaru3@clin.medic.mie-u.ac.jp

**Keywords:** low back pain, disc degeneration, platelet-rich plasma

## Abstract

*Background and Objectives:* Clinical studies of platelet-rich plasma (PRP) for the treatment of low back pain (LBP) have been reported; however, less is known about its long-term efficiency. *Materials and Methods:* This study was a long-term follow-up of a previous prospective clinical feasibility study for the use of PRP releasate (PRPr) to treat discogenic LBP patients. Among 14 patients, 11 patients were evaluated for a long-term survey. The efficacy was assessed by a visual analogue scale (VAS) for LBP intensity and the Roland-Morris Disability Questionnaire (RDQ) for LBP-related disability. Radiographic disc height was evaluated for seven patients. *Results:* Improvements in VAS and RDQ were sustained at an average of 5.9 years after the intradiscal injection of PRPr (*p* < 0.01 vs. baseline, respectively). Clinically meaningful improvements (more than 30% decrease from baseline) in VAS and RDQ were identified in 91% of patients at final survey. The radiographic measurement of disc height of PRPr-injected discs showed a mild decrease (13.8% decrease compared to baseline) during the average 5.9 years. *Conclusions:* The results of this study with a small number of patients suggest that the intradiscal injection of PRPr has a safe and efficacious effect on LBP improvement for more than 5 years after treatment. Further large-scale studies would be needed to confirm the clinical evidence for the use of PRPr for the treatment of patients with discogenic LBP.

## 1. Introduction

Low back pain (LBP) is a common musculoskeletal symptom from children to the elderly and is therefore a major public health problem globally [1]. A systemic analysis by the Global Burden of Diseases (GBD) study in 2019 reported that LBP is the leading cause of years lived with disability (YLDs) and proposed that providing preventive and curative interventions is highly recommended for reducing the future burden of LBP [2,3].

Recent epidemiological studies have shown evidence that intervertebral disc (IVD) degeneration is one of the major causes of LBP [4,5,6,7]; this is called ‘discogenic LBP’. Increased expressions of pro-inflammatory cytokines [8,9] and/or neurotrophic factors [10,11,12] that are found in degenerated human IVDs are considered to generate nociceptive pain. Furthermore, disc ruptures, especially in annulus fibrosus tissues, in degenerated human IVDs are associated with the presence of LBP [13,14,15,16]. Namely, pro-inflammatory stimuli and/or disc ruptures found in degenerated IVDs are considered to be responsible for the presence of discogenic LBP. Biologically, suppressing these inflammatory responses [17] and promoting tissue repair within degenerated/degenerating IVDs [18,19,20] would be essential treatments for discogenic LBP (see reviews in [21,22,23]).

Platelet-rich plasma (PRP) is autologous whole blood concentrated through centrifugation. Activated platelets release a vast majority of bioactive proteins, including growth factors and cytokines, which promote tissue repair and reduce inflammatory reactions [24,25]. Furthermore, autologous PRP provides no concern for the risk of disease transmission and immune rejection and has antibacterial properties [26,27]. Therefore, PRP has been widely used in the field of bone and joint disorders and soft tissue injuries [28].

The effect of PRP on IVD degeneration has been investigated in vitro and in animal models, and it is reported that PRP has significant reparative effects to counteract IVD degeneration (see review in [29]). A recent meta-analysis of clinical trials of intradiscal PRP injections for patients with discogenic LBP showed a beneficial effect on pain relief outcome [30]; however, further studies are needed to confirm the clinical evidence of PRP therapy for the treatment of LBP.

Cheng et al. [31] assessed the long-term effectiveness of intradiscal injections of PRP for discogenic LBP patients of a previous randomized controlled trial [32] and reported that PRP therapy showed clinically significant improvements in both pain and function at 5–9 years post-injection.

We conducted a preliminary clinical study to assess the effect of the releasate isolated from PRP (PRPr) from patients with discogenic LBP and showed its safety and preliminary analgesic effect [33]. A recent double-blind randomized controlled trial showed that the intradiscal injection of PRPr significantly improved the LBP intensity similar to that of glucocorticoid injections at eight weeks post-injection [34]; however, the long-term effect of the intradiscal therapy by PRPr remains unknown.

The purpose of this study was to evaluate the long-term efficacy of intradiscal injections of PRPr on LBP intensity, disability, and radiographic disc height in patients with discogenic LBP.

## 2. Materials and Methods

### 2.1. Study Design and Patients

This study was a long-term follow-up of a previous prospective clinical trial, primarily a safety assessment, conducted between April 2009 and March 2012 [33]. This long-term follow-up study was approved by the Clinical Research Ethics Review Committee of Mie University Hospital. Informed consent was obtained in written form or in the opt-out form on the website.

The patients who had a symptomatic degenerated disc(s) had received intradiscal injections of the releasate isolated from leukocyte-poor PRP. Inclusion criteria for the previous clinical trial [33] were being older than 18 years and having (1) chronic low back pain without leg pain for more than 3 months, (2) one or more lumbar discs (L3/L4 to L5/S1) with evidence of degenerative changes as per MRI (disc degeneration was defined as more than grade III by the Pfirrmann disc degeneration grade/classification system [35]), (3) maintenance of 50% or more normal disc height, and (4) at least one symptomatic disc confirmed using standardized provocative discography and/or disc block. Exclusion criteria included abnormal neurological symptoms (e.g., radiculopathy) with lumbar spinal stenosis or spondylolisthesis and inflammatory arthritis (e.g., discitis).

### 2.2. Preparation of PRP Releasate (PRPr)

PRP isolation was performed as previously reported [33]. In short, whole blood with an anti-coagulant was first centrifuged at 3000× *g* for 15 min at room temperature to form a buffy coat (BC) layer containing platelets and leukocytes. The BC layer was resuspended in 20 mL of plasma and then centrifuged at 180× *g* for 15 min to separate the platelets from leukocytes and residual red blood cells. The resulting supernatant was PRP. Autologous serum and 2% CaCl_2_ (Otsuka, Tokyo, Japan) was added to the PRP for clot (gel) formation. After incubation for 60 min at room temperature, the supernatant (releasate) was isolated from the PRP gel (PRPr) by centrifugation (3000× *g* for 5 min). The samples were kept at −20 °C until used.

### 2.3. Follow-Up Survey

The follow-up survey was conducted either by visiting the outpatient department or mail. The efficacy of this treatment was assessed by visual analogue scale (VAS) for LBP and the Roland-Morris Disability Questionnaire (RDQ) [36] for back-pain-related disability. LBP intensity was measured on VAS (0–100 mm), which is reliable, valid, and sensitive to change in the evaluation of pain intensity [37,38,39]. RDQ is a scale that allows the patients themselves to assess the degree of disability experienced during daily activities because of low back pain. There are 24 items that ask about the degree of disability experienced during daily activities such as standing, walking, sitting, getting dressed, and working [36,40,41].

Lateral lumbar spine radiographs of patients were taken centered on the L3 vertebrae in the standing position before and after treatment, as well as every second month until the end of the study and at follow-up survey. The anterior and posterior heights and the depths of the intervertebral discs were measured, and the disc height index (DHI) of target discs was calculated as previously reported [42]. The %DHI was calculated as the rate of change in DHI compared to the baseline ((DHI at follow-up–DHI at baseline)/DHI at baseline) × 100%.

### 2.4. Statistical Analysis

Time-dependent changes in %DHI were assessed for statistical significance by one-way repeated measures analysis of variance (ANOVA). VAS scores at each time-point against baseline were evaluated by a paired *t*-test. Temporal changes of RDQ against baseline were evaluated by the Friedman test, followed by a post hoc test by Wilcoxon signed ranks test. The data were expressed as mean ± standard deviation (SD). All the statistical analyses were performed using IBM Statistical Package for Social Sciences (SPSS) software (IBM Japan, Tokyo, Japan). The accepted level of significance was *p* < 0.05.

## 3. Results

### 3.1. Baseline Characteristics of Follow-Up Subjects

Among 14 subjects evaluated from the previous clinical study, VAS and RDQ data were obtained from 11 patients (Average age: 33.6 ± 8.2, 5 male and 6 female) (Table 1). The average follow-up period was 5.95 (±0.87) years (from 4.58 to 7.25). Ten patients received a single injection of PRPr, and one patient (#09) received a second injection of PRPr at three months after the first injection. The mean age at baseline was 34.4 (±8.6) years old (five male and six female). Among them, radiographic data were obtained from seven patients who agreed to visit the outpatient department (Average age: 33.7 ± 8.2, three male and four female). The targeted discs were L4/L5 (in 9 cases) and L5/S1 (in 2 cases). The MRI Pfirrmann disc degeneration grade was three in nine patients and grade 4 in two patients.

### 3.2. Assessment of Low Back Pain

**Figure 1:**
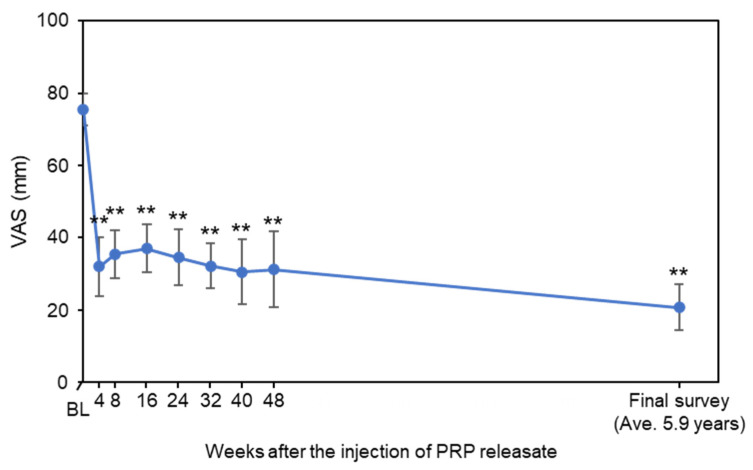
Change in visual analogue scale (VAS). Values are shown as mean ± standard error of the mean (SEM). ** *p* < 0.01 vs. baseline (BL).

VAS scores at baseline (75.5 ± 14.4) were decreased from 4 to 48 weeks after the injection; this was maintained at the final survey (20.7 ± 21.1, average 5.9 years from baseline) (Figure 1). VAS of each time point from 4 weeks to final survey was lower than that of the baseline (*p* < 0.01 vs. baseline, respectively) (Figure 1). Clinically significant improvement in pain (≥30% reduction in LBP VAS) was observed in 91% of subjects at the final survey (Table 2). Pronounced to complete pain relief (≥75% reduction in LBP VAS) was demonstrated in 7 of 11 subjects (64%) (Table 2).

**Figure 2:**
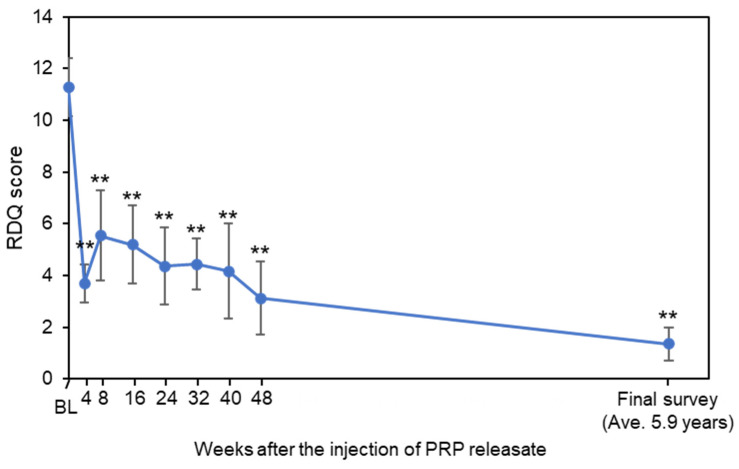
Change in Roland-Morris Disability Questionnaire (RDQ). Values are shown as mean ± standard error of the mean (SEM). ** *p* < 0.01 vs. baseline (BL).

RDQ at baseline (11.3 ± 3.7) was decreased from 4 to 48 weeks after the injection; this was maintained at final survey (1.4 ± 2.1, average 5.9 years from baseline, *p* < 0.01 by the Friedman test) (Figure 2). RDQ of each time point from 4 weeks to final survey was lower than that of baseline (4W-Final: *p* < 0.05, vs. baseline, respectively) (Figure 2). Clinically significant improvements in LBP-related QOL score (RDQ score, ≥30% reduction) were found in 10 subjects (91%) (Table 2). Pronounced improvement (75–100% reduction in RDQ) was demonstrated in 9 of 11 (82%) at final survey (Table 2).

### 3.3. Change in Disc Height

**Figure 3:**
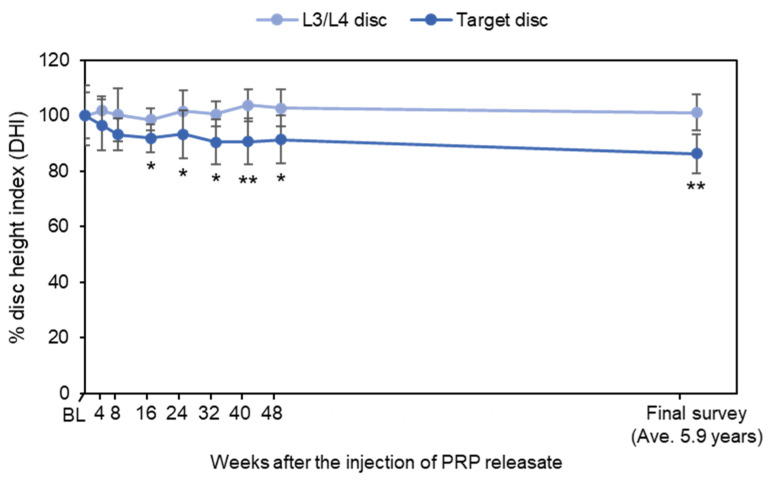
Change in disc height. Disc height was represented as the rate of change in disc height index (DHI) compared to the baseline (BL) (%DHI). Values are shown as mean ± standard error of the mean (SEM). * *p* < 0.05, ** *p* < 0.01 vs. L3/L4 discs.

Among 14 subjects evaluated from the previous clinical study, lumbar radiographs were obtained from seven patients. Repeated measurement of analysis of variance revealed that there were no significant time-dependent changes of %DHI (*p* = 0.26), although %DHI differed between L3/4 discs and target discs (*p* < 0.01) (Figure 3). There was no significant interaction between the disc level and time point (*p* = 0.07). A time point analysis revealed that %DHI of target discs at 16, 32, 40, 48 weeks and final follow-up was lower than that of the L3/L4 control discs (16, 32 and 48 weeks: *p* < 0.05; 40 weeks and final follow-up: *p* < 0.01, all vs. L3/L4 discs) (Figure 3).

## 4. Discussion

The results of this long-term follow-up survey showed that the LBP intensity scores (VAS), and LBP-related disability scores (RDQ) remained decreased for an average of 5.9 years after the intradiscal injection of PRPr. Clinically meaningful improvements of VAS and RDQ (more than 30% decrease from baseline [37]) scores were identified in 91% of subjects at final survey, even though the radiographic measurement of disc height of PRPr-injected discs showed a mild decrease (13.8% decrease compared to baseline) during the average 5.9 years.

Recently, Chen et al. [31] conducted a follow-up study that assessed back pain and physical function post-injection of PRP for patients with discogenic low back pain who were previously enrolled in a randomized control trial; they reported that significant improvements in pain and physical function were identified at 5–9 years post-injection. In accordance with the results of Cheng’s follow-up study, the results of our long-term survey showed preferable results on LBP and related disability after PRPr treatment. These results suggest the possibility that the intradiscal injection of PRP and/or activated PRPr have an encouraging safe and efficacious analgesic effect for more than 5 years after treatment.

Change in disc height, which reflects structural change of IVDs, is one of the basic clinical indications that represents the extent of IVD degeneration [42]. We have previously reported that the intradiscal injection of autologous PRPr restored disc height in the rabbit IVD degeneration model throughout 8 weeks after treatment [43]. However, the results of this long-term study revealed that disc height of PRPr-injected discs was mildly decreased (13.8% decrease) during an average of 5.9 years. Our previous population-based longitudinal study on lumbar disc height narrowing showed that disc height of the lumbar spine in the elderly population showed a 3.0% decrease for 6 years [42]. Compared to the natural history of the change in lumbar disc height, although the subjects’ ages were different, the results of this long-term study suggest that intradiscal injection existing PRPr would have no potential to suppress the progression of disc height narrowing of the degenerating IVDs. Multiple injections or quality modification of PRPr might be needed to gain the tissue remodeling effect on degenerating/degenerated IVDs.

Thus far, 12 clinical studies that assessed the effects of intradiscal injection of PRP for patients with discogenic low back pain have been reported [30]. Among them, PRP itself was directly injected into degenerated discs in 11 studies, except for the one preliminary study of a PRPr injection [29]. It is considered that the injected platelets in PRP would be endogenously activated by the tissue collagen within the degenerated discs, and then the bioactive proteins would be naturally released [44]. On the other hand, PRPr is the releasate that is isolated from exogenously activated platelets; therefore, PRPr does not contain residual platelets after activation. Namely, PRPr contains only the serum fraction with concentrated bioactive proteins. All the clinical studies did show positive effects on back pain reduction; hence it remains unknown whether PRP or PRPr is more effective for back pain patients. Activated PRPr treatment has an advantage on cryopreservation, sterilization at injection, though the preparation of PRPr needs the PRP activation process and the isolation process of the supernatant in aseptic condition, additionally. Future clinical study would be needed to compare the clinical efficacy of PRP and PRPr on discogenic low back pain patients.

It is well known that PRP and/or PRPr has a significant anti-inflammatory effect [29,45]. Pain reduction in the early treatment phase would be due to the anti-inflammatory effect of PRP or PRPr [29,33]. However, it remains unknown why the improved effect of PRP lasts a long time after the injection of PRP (or PRPr). Nevertheless, we confirmed the long-term effect of PRPr injection therapy on pain reduction for patients with discogenic LBP.

There were several limitations of this study. First, the sample size of patients was small. Therefore, an increased number of patients would be needed for the efficacy estimation of PRPr treatment. Second, among 14 patients in a previous prospective clinical trial, 11 patients were evaluated for the LBP intensity and disability, and 7 patients were available for lumbar radiographic evaluation (including one patient who received the additional PRPr injection) in this long-term follow-up study. Therefore, the attrition bias affecting the validity and reliability of the results [46] does exist in this study. Thirdly, MRIs were not taken for the subjects of the long-term follow-up survey; therefore, MRI evaluation would be needed to assess the extent of disc degeneration itself. Lastly, this study did not contain control subjects. To precisely evaluate the long-term efficacy of this treatment, a randomized control study would be needed.

## 5. Conclusions

This study first reported the long-term results of the intradiscal injection of PRPr for patients with discogenic low back pain. Our results showed that LBP remained improved, and activities of daily living (ADLs) were not impaired by LBP for an average of six years after PRPr injection. Furthermore, clinically meaningful improvements in LBP intensity (VAS) and LBP-related disability scores (RDQ) were observed in more than 90% of subjects at the final survey. On the other hand, the disc height of PRPr-injected discs was mildly decreased (13.8% decrease) during the average 5.9 years, suggesting that PRPr has no potential to induce a structural restoration of degenerated IVDs. However, this study did not set the control; hence, it cannot deny the possibility that the results of this follow-up study reflect the natural histology of degenerative disc disease itself. Since the number of patients was small in this clinical study, including attribution bias, a further large-scale study would be needed to confirm the clinical evidence of PRPr for the treatment of patients with discogenic LBP.

## Figures and Tables

**Table 1:** Patient characteristics (baseline).

Patient ID	Age	Gender	VAS	RDQ	Disc Level	MRI Phirmann’s Grade	X-ray at Final Follow-Up
#01	4X	M	70	11	L4/L5	III	−
#02	2X	F	80	12	L4/L5	III	+
#04	4X	M	80	8	L4/L5	III	+
#05	2X	F	80	17	L4/L5	IV	−
#06	3X	F	50	8	L4/L5	III	+
#07	2X	M	90	15	L5/S1	III	+
#08	4X	M	70	13	L5/S1	III	+
#09	2X	F	90	16	L4/L5	IV	+
#12	4X	M	90	22	L4/L5	III	+
#13	2X	F	80	10	L4/L5	III	−
#14	2X	F	50	6	L4/L5	III	−

Visual analog scale: VAS (100 mm), Roland-Morris Disability Questionnaire: RDQ.

**Table 2:** Pain relief (VAS) and functional improvement (RDQ) at final survey.

	≥30% Reduction	≥50% Reduction	≥75% Reduction
VAS	91% (10/11)	64% (7/11)	64% (7/11)
RDQ	91% (10/11)	91% (10/11)	82% (9/11)

Visual analog scale: VAS, Roland-Morris Disability Questionnaire: RDQ.

## Data Availability

The data presented in this study are available on reasonable request from the corresponding author.
